# Anti-inflammatory effects of vagus nerve stimulation in pediatric patients with epilepsy

**DOI:** 10.3389/fimmu.2023.1093574

**Published:** 2023-02-10

**Authors:** Supender Kaur, Nathan R. Selden, Alejandro Aballay

**Affiliations:** ^1^ Department of Molecular Microbiology & Immunology, Oregon Health & Science University, Portland, OR, United States; ^2^ Department of Neurological Surgery, Oregon Health & Science University, Portland, OR, United States

**Keywords:** neuroimmune, vagus nerve stimulation, seizure, tumor necrosis factor, cytokines, peripheral blood monocytes cells, insulin, blood glucose

## Abstract

**Introduction:**

The neural control of the immune system by the nervous system is critical to maintaining immune homeostasis, whose disruption may be an underlying cause of several diseases, including cancer, multiple sclerosis, rheumatoid arthritis, and Alzheimer’s disease.

**Methods:**

Here we studied the role of vagus nerve stimulation (VNS) on gene expression in peripheral blood mononuclear cells (PBMCs). Vagus nerve stimulation is widely used as an alternative treatment for drug-resistant epilepsy. Thus, we studied the impact that VNS treatment has on PBMCs isolated from a cohort of existing patients with medically refractory epilepsy. A comparison of genome-wide changes in gene expression was made between the epilepsy patients treated and non-treated with vagus nerve stimulation.

**Results:**

The analysis showed downregulation of genes related to stress, inflammatory response, and immunity, suggesting an anti-inflammatory effect of VNS in epilepsy patients. VNS also resulted in the downregulation of the insulin catabolic process, which may reduce circulating blood glucose.

**Discussion:**

These results provide a potential molecular explanation for the beneficial role of the ketogenic diet, which also controls blood glucose, in treating refractory epilepsy. The findings indicate that direct VNS might be a useful therapeutic alternative to treat chronic inflammatory conditions.

## Introduction

The nervous system plays an important role in the control of appropriate immune responses. A well-described neural-immune pathway concerns the activation of sensory vagal fibers by engagement of the IL-1β receptor, which results in the activation of anti-inflammatory mechanisms (1, 2). Thus, vagus nerve stimulation (VNS) could act as an anti-inflammatory therapy through the control of the neuro-immune axis. VNS plays a dual anti-inflammatory role *via* the efferent and the afferent fibers activating the cholinergic anti-inflammatory pathways (CAP) and the hypothalamic-pituitary-adrenal (HPA) axis (3). In this study, we describe recent findings regarding the transcriptional changes that take place upon chronic VNS in pediatric epilepsy patients.

Signals from the vagus nerve inhibit the production of cytokines and tumor necrosis factor (TNF) in monocytes and macrophages (4). Studies in different model systems have shown that stimulating the vagus nerve enhances the release of acetylcholine in the spleen and other tissues by activating choline acetyltransferase positive T cells (5). Studies also indicate that electrical stimulation of the vagus nerve can inhibit tumor necrosis factor (TNF) production in humans (6). However, the mechanism of such inhibition and its overall effects on immune cells remain unclear. To provide insights into the potential therapeutic implications of vagus nerve stimulation, we examined blood samples taken from a cohort of existing pediatric patients with localization-related epilepsy and ongoing VNS treatment. Pediatric epilepsy patients differ from adult patients regarding seizure semiologies and the prevalence of specific epilepsy syndromes and etiologies. For pediatric patients, the main goal of treatment is a reduction in seizure burden while minimizing treatment side effects (7). The treatment of drug-resistant epilepsy (DRE) is more challenging in children because of the additional impact of both seizure burden and treatment on neurodevelopment and risk for related permanent psychosocial impairment. Thus, the treatment of DRE is often more aggressive in children than in adults (8).

VNS has been used for decades in patients with epilepsy (9, 10). It has also shown anti-inflammatory properties, suggesting a role for VNS in the treatment of inflammatory disorders such as rheumatoid arthritis (RA) (6), inflammatory bowel disease and Crohn’s disease (3), lung injury and diabetes (11). Epilepsy is a comorbid disorder accompanied by cognitive, physiological, and neurological dysfunction. Epilepsy is characterized by recurring seizures due to excessive neuronal function resulting from disruption of neuronal activity (12). According to a 2006 World Health Organization (WHO) report, 2.4 million cases of epilepsy are diagnosed annually. The Global Burden of Disease Study of 2016 indicates that more than 50 million people have epilepsy worldwide (13). Although there are many underlying mechanisms responsible for epilepsy, the actual cause of the disease is still unknown. Standard therapies, including anti-epileptic drugs (AEDs), only help manage the disease but do not cure it. In addition, existing medications have severe side effects and significantly affect development, quality of life, and social functions (14). Treatment for epilepsy in its early stages is critical as uncontrolled seizures can damage the brain and reduce the ability to think and learn. Treatment also lowers the risk of sudden death during or after a seizure, but in about one-third of patients, seizures cannot be controlled with medication alone. It has been reported that among 146 patients treated with VNS, more than 90% had fewer seizures, 50% had shorter seizures, and almost 75% needed less medication (15).

To explore immune pathways that VNS may control, we examined the genome-wide gene expression changes that occur in human peripheral blood monocyte cells (PBMCs) from epileptic patients treated with VNS. PBMCs are a variety of specialized immune cells in the human body and are used for the immune response to infections and the development of immunotherapeutic strategies (16). We compared the gene expression changes in epileptic patients treated with VNS with the changes observed in an untreated control group. For transcriptional profiling, we used the peripheral blood mononuclear cells (PBMCs) isolated from whole blood samples from both groups. Our study showed that VNS inhibits several immune pathways in human PBMCs.

## Materials and methods

### Epileptic patients treated with anticonvulsants or anticonvulsants and VNS

To determine whether VNS controls inflammation in humans and genome-wide changes in gene expression, we studied pediatric epilepsy patients (≥5 to ≤18 years of age) with a localization-related epilepsy diagnosis. These patients were treated with anticonvulsant medication by their standard of care provider. For this study, there were two groups of 4 patients each (**Table 1**):

**Table 1:** A comparison of vagal nerve treated (n =4) and not treated (n = 4) seizure groups.

Group 1 (Treated)	Group 2 (Control)
Seizures	Seizures
Anticonvulsants	Anticonvulsants
Vagal Nerve Stimulator	No Vagal Nerve Stimulator

Both the treatment group and control group included 2 female and 2 male patients, ranging in age at the time of venipuncture from 8 to 17 years (treatment group) and 6 to 18 years (control group). Age at epilepsy onset was also similar between the groups, ranging from 4 months to 7 years in the treatment group and 2 months to 9 years in the control group. Patients in the treatment group were on 1 to 4 (median 2) medications, and in the control group, 1 to 3 (median 2) medications. One treatment group and two control group patients suffered from partial epilepsy, with the remainder in both groups suffering from primary or secondary generalized epilepsies. Intellectual delay and/or autism spectrum disorders were present in 3 patients in each group. The seizure frequency varied between multiple seizures per day and seizures approximately monthly, and was balanced between the two groups.

In each case, the VNS device used was the model 105 generator and 304 lead from Cyberonics, Inc., Houston, USA. The 4 treatment subjects had been treated continuously for 3 to 6 years with settings at the time of the study venipuncture ranging from 1.75 to 2.25 mA, 20 to 30 Hz, 250 to 500 microseconds square wave pulse, and off time from 1.8 to 5 minutes, with an on time in each case of 30 seconds.

After study consent was obtained, venous blood was drawn (3 mL/Kg up to 10 mL) one time during a routine clinic visit by a licensed nurse practitioner. Medical records were also collected for each subject and saved to a secure REDCap database.

### Study population

Our study included a pediatric population. Pediatric patients more commonly suffer from extra-temporal epilepsy treated by VNS and are more commonly affected by epilepsies with an inflammatory component. There were certain inclusion and exclusion criteria.

#### Inclusion criteria

(1) Positive diagnosis of medically refractory epilepsy, defined as focal seizures that are not controlled despite the use of two anti-seizure medications at correct doses.
(2) Patients aged ≥5 to ≤18 years
(3) Patient is neither febrile nor has any type of infection at the time of the visit
(4) Currently taking antiepileptic medications

#### Exclusion criteria

(1) Presence of a feeding tube or tracheotomy
(2) Presence of any other surgical implants
(3) Patient is febrile or has any type of infection at the time of the visit
(4) Patients aged < 5 years or > 18 years

### Recruitment methods

Samples were collected at Oregon Health & Science University (OHSU). Subjects were identified from the study team’s clinical practice on an ongoing basis, and recruitment was done during a Neurology clinic visit.

### Consent process

The study was approved by the Institutional Review Board. All procedures were explained to the child and parent during their clinic visit. We explained the study procedures involved (an extra blood draw), along with the subject’s rights within the study. We followed OHSU guidelines for collecting assent, including when children cannot provide consent due to cognitive impairment or due to young age. Consent was obtained from at least one parent if the child cannot provide consent, as per OHSU guidelines. If the child or the parent were not willing to participate, they were not enrolled in the study. We tried to minimize coercion by always reiterating that they are not under any obligation to participate in this study and that their care will not be affected. We used a short form and an OHSU translator if the potential subject had limited or no proficiency in English.

### Separation of peripheral blood mononuclear cells (PBMCs)

Fresh blood samples were used to ensure high recovery, purity, and viability of the isolated mononuclear cell fractions. Sample preparation was done at 18°C to 20°C.To a 15 ml centrifuge tube, there was an addition of 10 ml of defibrinated or anticoagulant treated blood. Next, the blood was transferred to 50 ml and added an equal volume of Phosphate buffered saline (PBS pH 7.4). After that, blood and buffer were mixed by inverting the tube several times or by drawing the mixture in and out of a pipette. To the centrifuge tube Ficoll-Paque PREMIUM (GE Healthcare) 3 ml was carefully layered and the diluted blood sample was added avoiding the mixing of the Ficoll-Paque and the diluted blood sample. Then, the centrifugation was done at 400 x g for 30 minutes. After centrifugation, the upper layer containing plasma and platelets was removed using a sterile pipette, leaving the layer of mononuclear cells undisturbed at the interface. The layer of mononuclear cells was transferred to a sterile centrifuge tube using a sterile pipette, followed by the addition of at least 3 volumes (approximately 6 ml) of balanced salt solution. Cells were suspended gently by drawing them in and out of a pipette, followed by centrifugation at 400 to 500 × g for 15min. The supernatant was discarded, and the mononuclear cells were again suspended in 6-8 ml of the balanced salt solution, followed by centrifugation at 500xg for 10 min. The supernatant was discarded, and the cell pellet was suspended in TRIzol (Life Technologies, Carlsbad, CA) and stored at -80oC for RNA isolation.

### RNA isolation and sequencing

Total RNA extraction of both control and treated samples was done using the RNeasyPlus Universal Kit (Qiagen, Netherlands) following the manufacturer’s protocol. The residual genomic DNA was removed using TURBO DNase (Life Technologies, Carlsbad, CA). Isolated RNA sample quality was assessed by High Sensitivity RNA Tapestation (Agilent Technologies Inc., California, USA and quantified by Qubit 2.0 RNA HS assay (ThermoFisher, Massachusetts, USA). Ribosomal RNA depletion was performed with Ribo-Zero Plus rRNA Removal Kit (Illumina Inc., California, USA). Samples are then randomly primed and fragmented based on the manufacturer’s recommendation. The first strand is synthesized with the Protoscript II Reverse Transcriptase with a longer extension period, approximately 30 minutes at 42°C. All remaining steps for library construction were used according to the NEBNext^®^ Ultra™ II Directional RNA Library Prep Kit for Illumina^®^ (New England BioLabs Inc., Massachusetts, USA). The final libraries quantity was assessed by Qubit 2.0 (ThermoFisher, Massachusetts, USA), and quality was assessed by TapeStation HSD1000 ScreenTape (Agilent Technologies Inc., California, USA). The average size of the final library was about 350bp with an insert size of about 200bp. Illumina^®^ 8-nt dual-indices were used. Equimolar pooling of libraries was performed based on QC values and sequenced on an Illumina^®^ NovaSeq S4 2x150 (Illumina, California, USA) with a read length configuration of 150 PE for 80M PE reads per sample (40M in each direction). Library preparation was done using NEBNext Ultra II RNA (Directional) with RiboZero Plus, and sequencing was performed using Illumina NovaSeq S4 2x150 at the Novogene Genomic Services & Solutions Company, USA.

### Gene expression analysis

For the study of changes in gene expression at the whole genome level, RNA-Sequencing was used. Samples of epilepsy patients VNS treated and non-treated were obtained and sequenced. The data was analyzed using the bioinformatics tool Galaxy. The sequenced reads from the 4 control and 4 treated samples obtained were mapped to the Human reference genome “Genome Reference Consortium Human Build 38 patch release 13” (GRCh38.p13) using the aligner STAR. For sequencing depth, RNA composition counts were normalized across all samples. Differential gene expression analysis was then performed on normalized samples using DESeq2. Genes exhibiting at least two-fold change and a false-discovery rate (FDR) of 0.05 were considered differentially expressed. Analysis for gene enrichment, gene ontology (GO), or biological processes was performed using the Database for Annotation, Visualization, and Integrated Discovery (DAVID) (david.abcc.ncifcrf.gov/).

## Results

To determine the effect of VNS on genome-wide changes in gene expression that takes place in human peripheral blood monocyte cells (PBMCs) in pediatric epilepsy patients, we studied four patients implanted with a VNS device and four patients without a VNS device. Peripheral blood was collected, and PBMCs were collected. Total RNA was isolated from the PBMCs, followed by RNA-sequencing to study the transcriptional changes that take place during VNS. Detailed information on the genes shown to be differentially expressed, including their respective fold change and p-values, can be found in **Table S1**. For RNA-seq analyses, treated and non-treated samples were considered as biological replicates of the treated versus control group using the bioinformatics tool Galaxy (17). The number of genes significantly upregulated or downregulated more than 2-folds were 180 and 563, respectively (**Tables S2, S3**).

**Figure 1:**
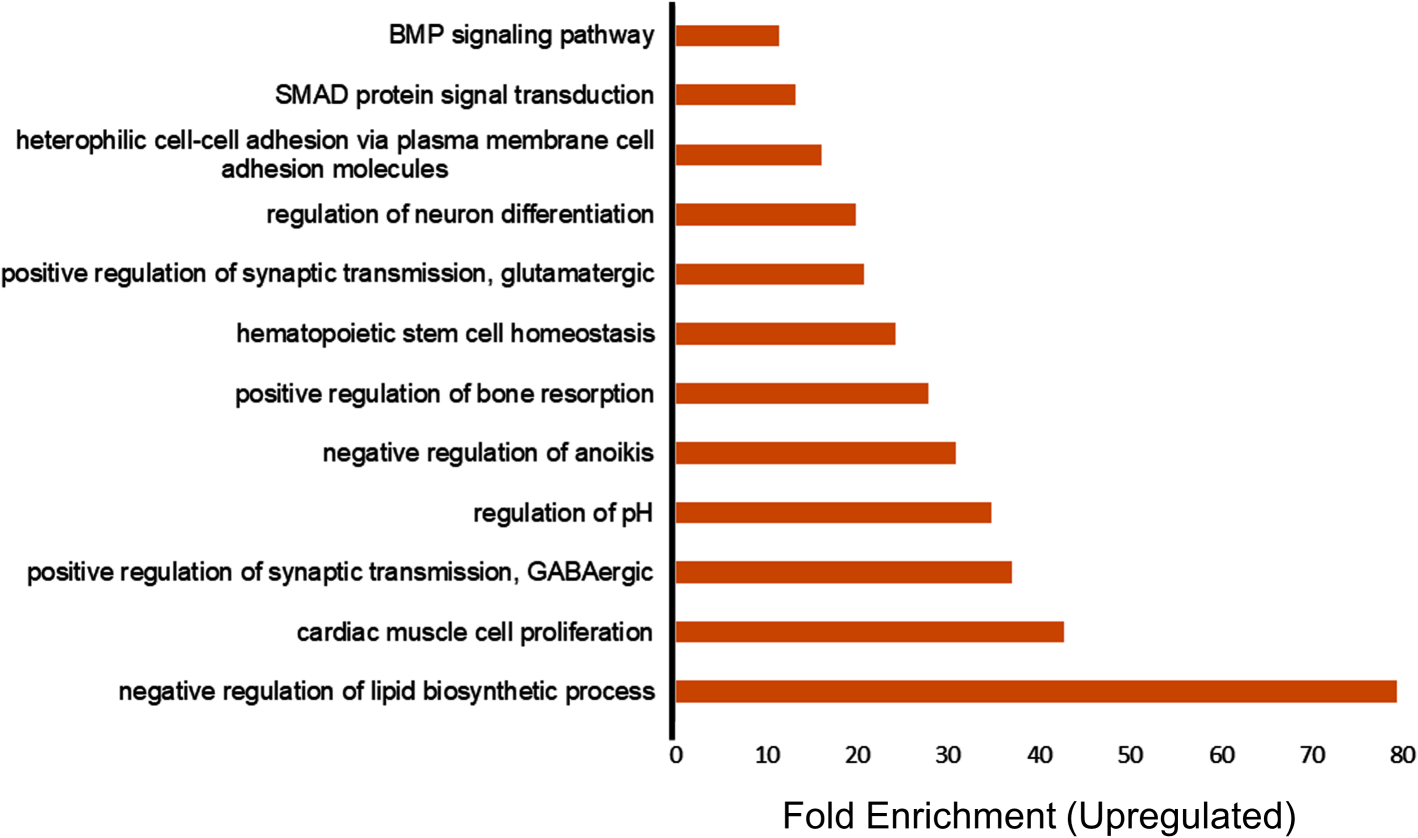
Transcriptome profiling of upregulated genes in patients treated with vagus nerve stimulation. Gene ontology (GO) enrichment analysis of upregulated genes in the patients treated with VNS vs. non- treated with P value of less than 0.05 and fold change more than 2 are included in the figure. The bars represent the different GO categories ordered by enrichment fold change shown on the x axis.

The upregulated and downregulated genes were analyzed separately for gene ontology analysis (GO) and functional annotation clustering using the bioinformatics tool DAVID (18). The upregulated genes showed significant enrichment of the GO categories associated with the negative regulation of lipid biosynthetic process, cardiac cell proliferation, positive regulation of gamma-aminobutyric acid (GABA) synaptic transmission, positive regulation of glutamatergic synaptic transmission, and regulation of neuron differentiation (**Figure 1**). We also found that VNS affected lipid biosynthetic pathways, which is consistent with previous studies in rats showing that VNS has a significant effect on lipid composition (19). The enrichment of the cardiac cell proliferation we observed is also consistent with previous studies indicating that VNS can be used as a potential therapy for various cardiovascular diseases (20). Furthermore, the enrichment of the synaptic transmission GABA GO category in PBMCs suggests that VNS upregulates GABA signaling in general, as it also increases levels of GABA in the cerebrospinal fluid of VNS patients (21).

**Figure 2:**
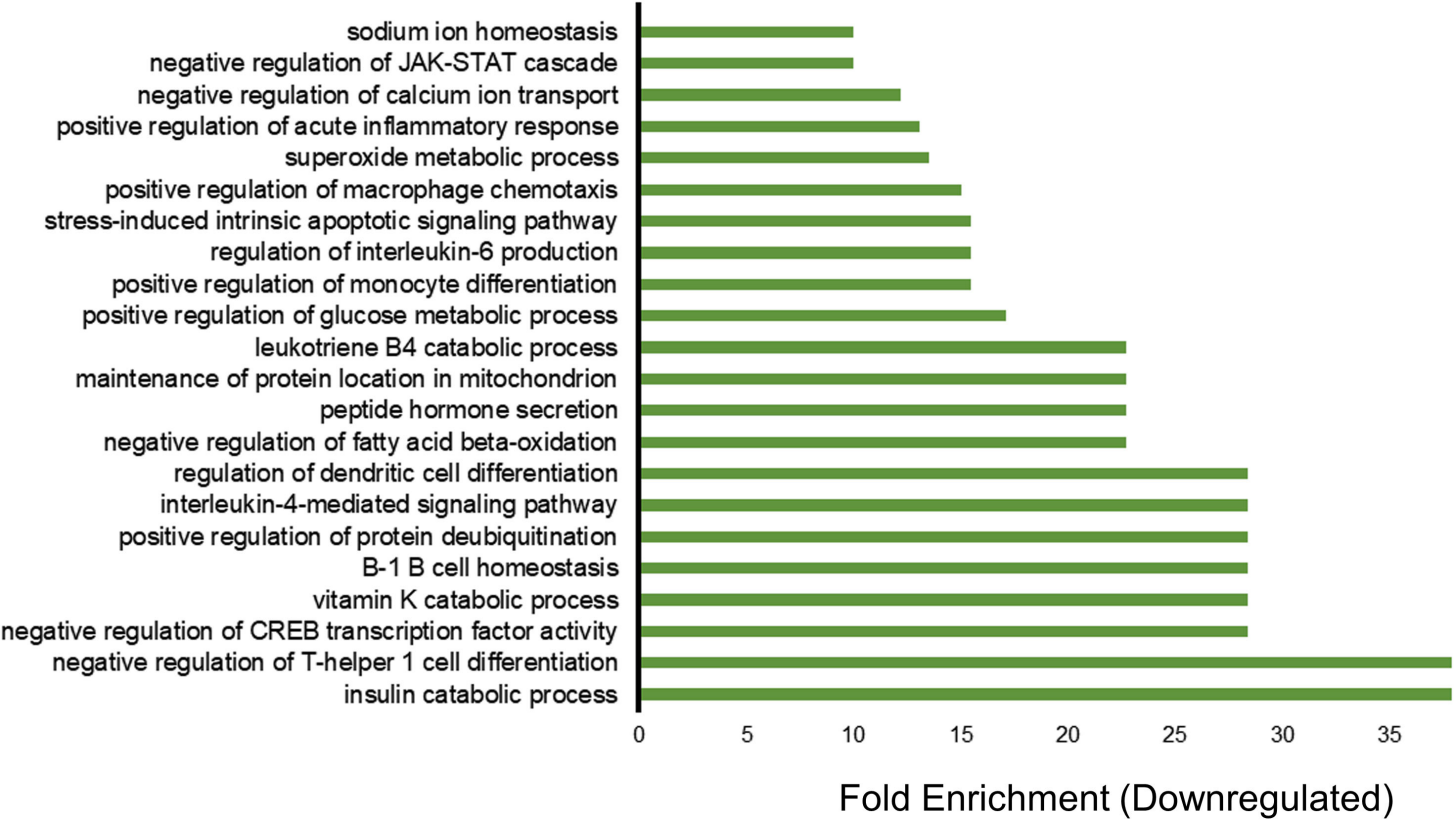
Transcriptome profiling of down-regulated genes in patients treated with vagus nerve stimulation. Gene ontology (GO) enrichment analysis of downregulated genes with P value of less than 0.05 and fold change of less than 0.5 are included in the figure. The bars represent the different GO categories ordered by enrichment fold change shown on the x axis. The category with the highest fold enrichment is at the bottom of the graph, while the lowest one is at the top of the graph.

For downregulated genes, enriched GO categories included insulin catabolic processes, negative regulation of T-helper 1 cell differentiation, negative regulation of CREB transcription factor activity, B-1 B cell homeostasis, positive regulation of protein de-ubiquitination, and cytokine interleukin IL-4 (**Figure 2**). The downregulation of the insulin catabolic process would result in more sugar storage and less circulating blood glucose. Interestingly, a ketogenic diet, which also controls blood glucose, is the most effective and frequent non-pharmacological treatment for refractory epilepsy (20, 21). These findings indicate that vagus nerve stimulation has the potential to modulate blood glucose and overall inflammation. Thus, VNS might be a useful therapeutic for inflammatory conditions and autoimmune diseases where excessive activation of the immune system can be deleterious or have fatal effects.

**Figure 3:**
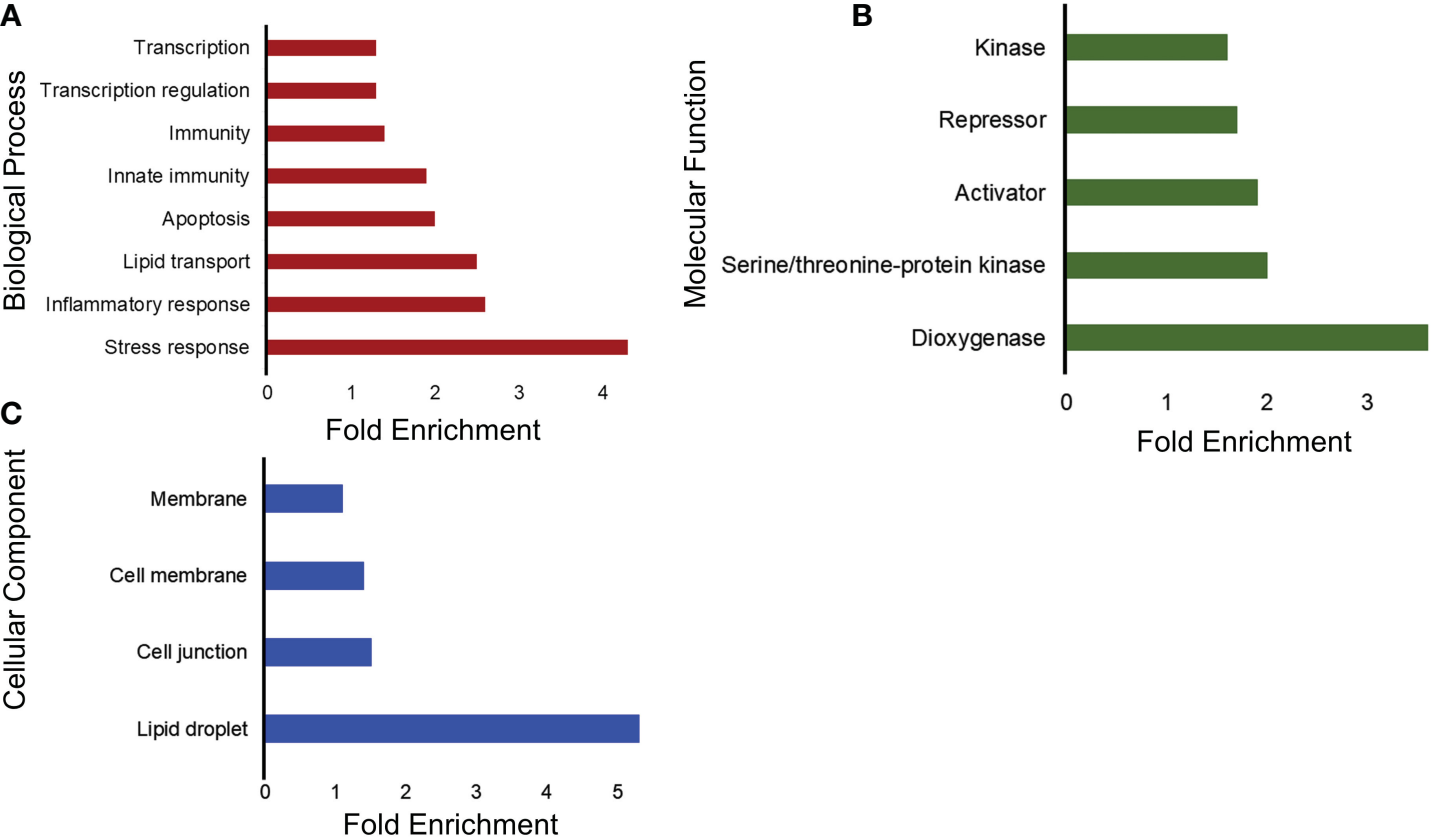
Functional annotation analysis of downregulated genes into three main categories using DAVID. **(A)** Representative bar graphs showing the significantly enriched genes associated with the biological process. The vertical lines represent the different biological processes involved and the horizontal and the length represent the fold enrichment. **(B)** Representative bar graphs showing the significantly enriched genes associated with the activities at the molecular level. The y-axis represents the different molecular functions ordered with the fold enrichment on the x-axis. **(C)** Representative bar graphs showing the significantly enriched genes associated with the cellular structures in which a gene functions and is termed as the cellular component. The y-axis represents the different cellular components ordered with the fold enrichment on the x-axis.

After the GO analysis for gene function, we analyzed the upregulated and downregulated genes for functional annotation, which describes the biological identity of genes in terms of biological roles, molecular function, cellular location, and expression. For the upregulated genes, the biological process involved was cell adhesion, and the cellular component was extracellular matrix. For molecular function, there was no significant enrichment. Moreover, the down-regulated genes showed significant enrichment in various terms, including molecular function, cellular component, and biological process. As shown in **Figure 3A**, there was a downregulation of genes related to stress, inflammatory response, and immunity. In terms of molecular function, genes encoding proteins with dioxygenase and kinase activity were downregulated (**Figure 3B**). The cellular component involved genes related to lipid droplets, cell junctions, and membranes (**Figure 3C**).

**Figure 4:**
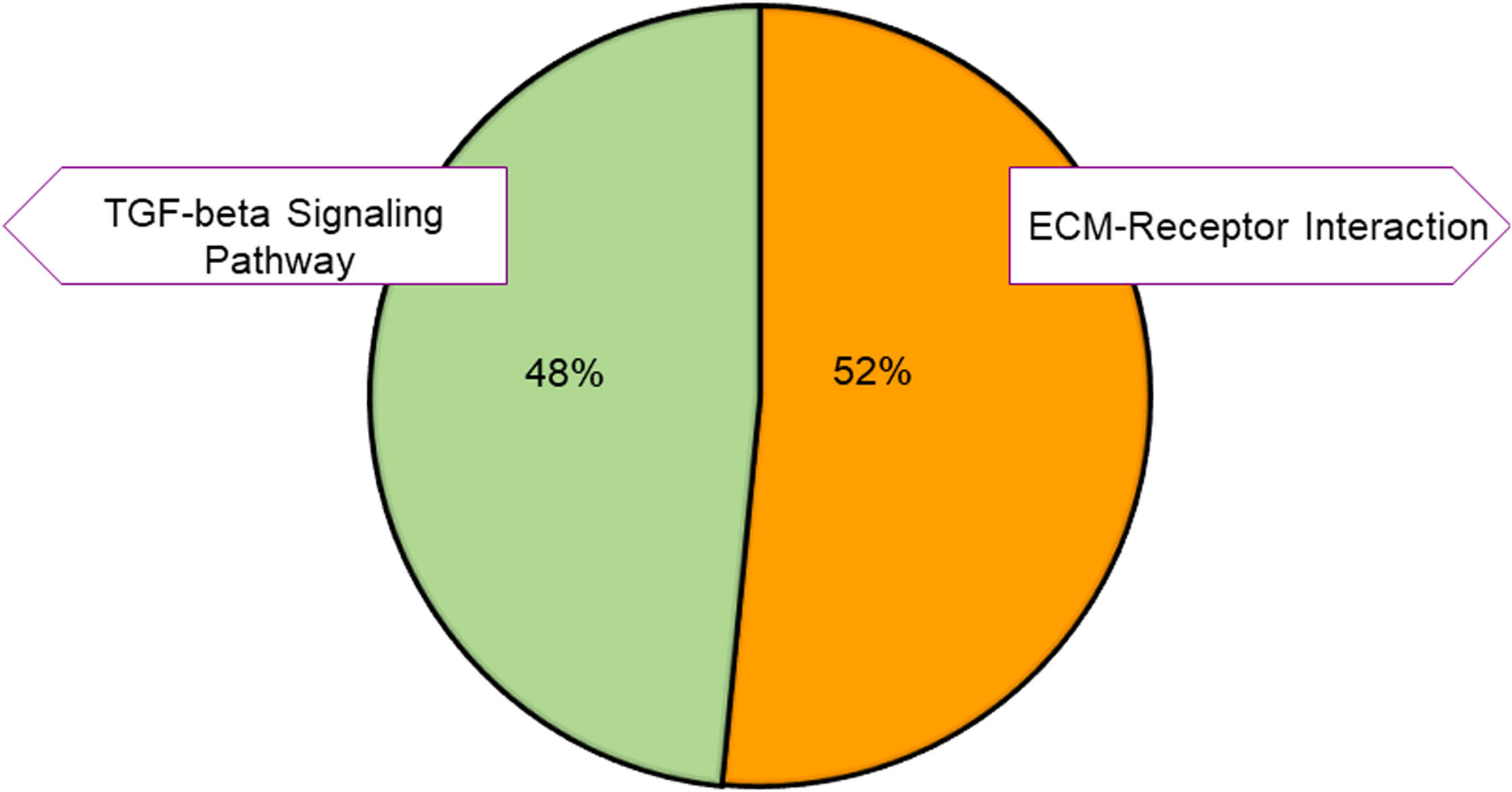
KEGG pathway classification of the upregulated genes. Pie charts showing the distribution of the KEGG pathways for the upregulated genes. The pathways were obtained by using DAVID. Different colors represent different pathways, and the percentage numbers represent the fold enrichment of the genes associated with that pathway.

To further identify different pathways activated by VNS, we used the Kyoto Encyclopedia of Genes and Genomes (KEGG). KEGG is a collection of databases of biological pathways related to diseases, chemical substances, and drugs (22, 23). As shown in **Figure 4**, the enriched pathways for the upregulated genes were related to the extracellular matrix receptor (ECM-receptor) interaction and the transforming growth factor-beta (TGF-beta) signaling pathways. The enriched TGF-beta signaling pathway has been reported to play an important role in cell growth, apoptosis, migration, and immune response (24). Studies have shown that dysregulation of TGF-beta signaling can cause various diseases, including leukemia, impaired wound healing, and neurodegenerative conditions (25, 26). The loss of TGF-beta signaling promotes tumorigenesis *via* suppression of the immune system (27), and the production of the TGF-beta1 isoform from the immune cells has an anti-inflammatory function (28, 29).

**Figure 5:**
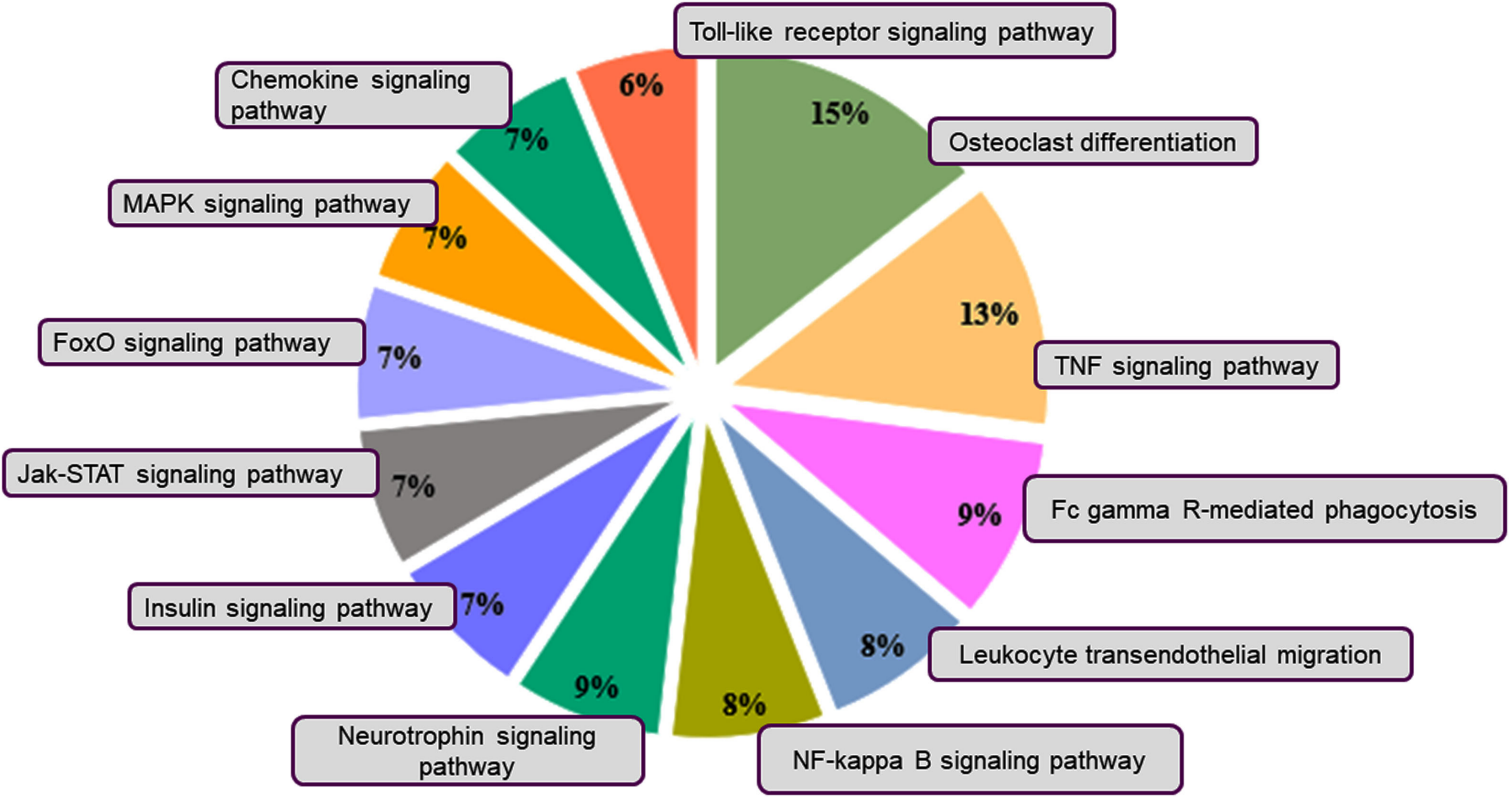
KEGG pathway classification of the downregulated genes. Pie charts depict the distribution of the KEGG pathways for the downregulated genes. The pathways were obtained by using DAVID. Different colors represent different pathways, and the percentage numbers represent the fold enrichment of the genes associated with that pathway.

For the down-regulated genes, the enriched pathways were the TNF signaling, JAK-STAT signaling, FoxO signaling, MAPK signaling, Chemokine signaling, and Toll-like receptor signaling pathways (**Figure 5**). The tumor necrosis factor (TNF) is an important regulator of immune responses, and inhibition of TNF is reported in the treatment of many inflammatory diseases, including inflammatory bowel disease and rheumatoid arthritis in mouse models and patients (30–33). The JAK/STAT pathway also modulates signals to maintain homeostasis in inflammatory conditions and is an important mediator in diseases like sepsis, cancer, diabetes, and autoimmunity (34). The KEGG pathway enrichment analysis further confirmed that there was downregulation of the immune-related pathways and upregulation of the anti-inflammatory pathways.

## Discussion

Vagus nerve stimulation (VNS) consists of the use of implanted or noninvasive devices that can modulate the activity of the nerve by delivering intermittent, controlled electrical impulses. The implanted device stimulates the brainstem *via* the vagus nerve and, *via* the brainstem, reaches different areas of the brain. VNS usage is approved for epilepsy, depression, and stroke rehabilitation, where traditional treatments have not been successful. Studies have shown that VNS significantly reduces the number of seizures, but the precise mechanism of action of VNS remains obscure.

Here, we studied the effect of VNS on PBMCs isolated from pediatric patients with localization-related epilepsy. Although epilepsy affects adults and children, the effect is more severe in the brain of pediatric patients. Because the pediatric brain is developing, recurrent seizures can permanently damage it, leading to changes in behavior, mental ability, and even the shape of the brain. Seizures also affect neural circuits leading to motor problems and life-long mental problems. Through transcriptomic studies, we found that VNS transcriptionally influences a myriad of pathways in pediatric patients. Our results showed that VNS treatment upregulated genes corresponding to the GABA and glutamate GO category. Studies have shown that balance between the two neurotransmitters helps to control overall excitation levels in the brain as GABA is inhibitory and glutamate is excitatory in action (35). There was upregulation of the TGF-beta signaling pathway in the VNS treated, and studies have shown that TGF-β is a protective anti-inflammatory cytokine that plays an important role in the suppression of inflammation (35, 36). Additionally, genes related to neural apoptosis and inflammatory responses were downregulated in PBMCs from patients treated with VNS. Similarly, there was also downregulation of the important immune signaling pathways, including TNF, JAK-STAT, FoxO, Chemokine, Toll-like receptor, and MAPK. There was downregulation of the tumor necrosis factor (TNF) pathway, which is involved in the regulation of immune responses in diseased and healthy organisms. TNF plays crucial role in immune response development and proper functioning by mediating the signaling associated with cell survival and cell death (37). The inhibitors of TNF-α can be used to treat inflammatory diseases, including inflammatory bowel disease and rheumatoid arthritis (30–33). Also, there was downregulation of the JAK/STAT pathway that modulates signals to maintain homeostasis in inflammatory conditions like sepsis, cancer, diabetes, and autoimmunity (34). Interestingly, results from previous studies have shown a similar inhibition of TNF in rheumatoid arthritis patients treated with VNS further strengthening our results (6).

In conclusion, our results provide evidence that VNS affects different pathways in PBMCs and strengthens the notion that VNS can control the neural-immune axis. Medically refractory epilepsy results, in part from abnormal brain activity (38, 39) as well as altered immune responses (40–42). In addition to its well documented effects on neural activity, VNS treatment palliate medically refractory epilepsy by regulating immune-mediated pathways. Specifically, our results demonstrate that VNS results in a decline in the production of pro-inflammatory cytokines. In the future, it will be interesting to further delineate the VNS-mediated neuro-immune regulation through cytokine profiling and potentially through the identification of relevant modulatory effects at the brain cortical or nuclear level. Finally, use of a transcriptome-wide approach may help us similarly identify other pathways related to the broad and expanding use of VNS for other disease states and therapeutic purposes.

The limitation of the study was the small sample size of control and treated patients. Thus, future studies with a larger sample size may be necessary to bring out a generalized conclusion on the entire population. It will also be informative to assess pre-PBMCs with serial PBMC after VNS treatment.

## Data availability statement

The datasets presented in this study can be found in online repositories. The names of the repository/repositories and accession number(s) can be found below: GSE210043 (GEO).

## Ethics statement

Ethical review and approval was not required for the study on human participants in accordance with the local legislation and institutional requirements. Written informed consent to participate in this study was provided by the participants’ legal guardian/next of kin.

## Author contributions

SK, NS, and AA conceived and designed the experiments. SK performed the experiments. SK and AA analyzed the data, and SK, NS, and AA wrote the paper. All authors contributed to the article and approved the submitted version.
